# Uncovering Gaps in Obesity Medicine Competencies: Insights from Ten U.S. Medical Schools

**DOI:** 10.1007/s40670-026-02646-2

**Published:** 2026-01-24

**Authors:** Magdalena Pasarica, Robert F. Kushner, Angelina V. Leary, Monica Agarwal, Dee-Ann Carpenter, Colleen Croniger, Ricardo Correa, Eduardo Grunvald, Mark S. Johnson, Julie Loza, Andrew Mock, Amber Olson, Jonathan Purnell, Kim Pfotenhauer, Virginia Uhley, Sadie Trammell Velasquez, Amanda Velazquez, B. Gabriel Smolarz

**Affiliations:** 1 Department of Medical Education, University of Central Florida College of Medicine, Orlando, FL USA; 2 Departments of Medicine and Medical Education, Northwestern University Feinberg School of Medicine, Chicago, IL USA; 3 Department of Psychology, University of Central Florida College of Sciences, Orlando, FL USA; 4 Departments of Medicine and Medical Education, The University of Alabama at Birmingham, Birmingham, AL USA; 5 Department of Native Hawaiian Health and Office of Medical Education, University of Hawaiʻi at Mānoa John A. Burns School of Medicine, Honolulu, HI USA; 6 Case Western Reserve University School of Medicine, Cleveland, OH USA; 7 Department of Endocrinology and Metabolism, Medical Specialty Institute, Cleveland Clinic, Cleveland, OH USA; 8 Department of Medicine, University of California San Diego School of Medicine, La Jolla, CA USA; 9 Department of Community and Family Medicine, Howard University, Washington, DC USA; 10 Department of Family and Community Medicine, University of Illinois College of Medicine at Chicago, Chicago, IL USA; 11 Department of Preventive Medicine, Loma Linda University School of Medicine, Loma Linda, CA USA; 12 Department of Medicine, Oregon Health & Science University, Portland, OR USA; 13 Michigan State University College of Osteopathic Medicine, East Lansing, MI USA; 14 Department of Foundational Medical Studies, Oakland University William Beaumont School of Medicine, Rochester, MI USA; 15 Department of Medicine, UT Health San Antonio Joe R. and Teresa Lozano Long School of Medicine, San Antonio, TX USA; 16 Departments of Medicine and Surgery, Cedars-Sinai Medical Center, Los Angeles, CA USA; 17 Novo Nordisk Inc, Plainsboro, NJ USA; 18 Division of Endocrinology, Metabolism and Molecular Medicine, Northwestern University Feinberg School of Medicine, 645 N Michigan Ave, Ste 530, Chicago, IL 60611 USA

**Keywords:** Obesity, Undergraduate medical education, Obesity medicine curricula, Obesity competencies

## Abstract

Competency-based obesity medicine education is critically needed in undergraduate medical training, as emphasized by the Association of American Medical Colleges. Using a competency-based framework, we identified key gaps in obesity education across ten diverse U.S. medical schools. Each institution formed a working group to conduct a gap analysis of the curriculum based on 32 obesity medicine competencies recognized by the obesity medicine fellowship council. The least-addressed domains were Practice-Based Learning and Improvement and Systems-Based Practice, accounting for nine of the ten least-covered competencies. Additional gaps were noted in clinical skills and bias awareness. Most schools were relying on passive teaching methods and limited assessment. Findings highlight opportunities to develop shared, scalable resources to strengthen and standardize obesity medicine education nationwide.

**Supplementary Information:**

The online version contains supplementary material available at 10.1007/s40670-026-02646-2.

## Background

The American Medical Association (AMA) has called for the integration of obesity education into medical school curriculum, recognizing the need to better prepare future physicians to manage this prevalent and complex disease [1]. Similarly, the Association of American Medical Colleges (AAMC) has emphasized strengthening competency-based education in the context of chronic diseases such as obesity—highlighting the importance of developing comprehensive training that extends beyond traditional nutrition education to encompass prevention, diagnosis, and long-term management [2]. Furthermore, current medical providers note that more training in obesity medicine is needed [3]. This focus highlights the urgency of preparing future physicians to address obesity, one of the most common noncommunicable diseases worldwide [4] with significant morbidity and mortality and linked to over 200 medical complications and comorbidities, including type 2 diabetes, heart disease, metabolic dysfunction-associated steatotic liver disease, and some forms of cancer [5].

The AAMC recommends conducting competency-based gap analyses and implementing targeted educational interventions that can be shared, refined, and scaled nationally to promote consistent, evidence-based training across medical schools [2]. Following this approach, we aim to identify the most critical gaps in obesity education across U.S. medical schools. Our analysis uses as a framework the 32 recognized obesity medicine competencies [6], organized into six domains based on the Accreditation Council for Graduate Medical Education (ACGME) framework, which defines the essential knowledge, skills, and attitudes required for effective patient care [7]. Our long-term goal is to inform the development of shared, scalable resources and to advance the integration of comprehensive, evidence-based obesity medicine education nationwide.

## Activity

Medical schools from across the US were recruited for participation in this gap analysis study by advertising in The Obesity Society electronic newsletter, the AAMC communities, and the Society of Teachers of Family Medicine electronic listserve. Nineteen schooled applied, and 10 were selected based on expertise, resources and commitment to the project, and most importantly, school diversity. Each school strived to establish a task force to include leadership from academic affairs (associate dean, chair, course director, chair of curriculum committee, director of instructional design and assessment), clinical affairs (section head, director of obesity center) and relevant stakeholders, such as residents, students, faculty and staff. The task force worked collaboratively to perform a structured audit of the existing undergraduate medical education curriculum for obesity medicine with the goal of identifying consistent gaps. Data was reported in an electronic survey using Qualtrics. Curriculum coverage for each of the 32 competencies was rated via a 4-point Likert scale, with 0 indicating not addressed, 1 indicating needs substantial improvement, 2 indicating needs some improvement, and 3 indicating adequate. Next, the teaching and assessing methods were indicated for each competency.

Data is presented using descriptive statistics. The study was reviewed by the Northwestern University Institutional Review Board, which determined that the activity is not research involving human subjects.

## Results and Discussion

Selected schools represented varied regions of the US, had a diversity of student populations, and conferred a Doctor of Medicine or Doctor of Osteopathic Medicine degree (Table 1). This varied representation in school size and funding, diversity in student population, and geographic region was important to generate and assess curricula that would be generalizable to the wide range of medical schools throughout the US.

**Table 1:** Characteristics of the medical schools

School name	City, state	Degree granted	Private/public	Total students enrolled, No.
Case Western Reserve University School of Medicine	Cleveland, OH	MD	Private	736
Cleveland Clinic Lerner College of Medicine	Cleveland, OH	MD	Private	128
Howard University College of Medicine	Washington, DC	MD	Private (historically Black college or university)	497
Loma Linda University School of Medicine	Loma Linda, CA	MD	Private	700
Michigan State University College of Osteopathic Medicine	East Lansing, MI	DO	Public	1200
Oakland University William Beaumont School of Medicine	Rochester, MI	MD	Private	500
University of California San Diego School of Medicine	San Diego, CA	MD	Public	575
University of Hawaiʻi John A. Burns School of Medicine	Honolulu, HI	MD	Public	289
University of Illinois College of Medicine	Chicago, IL	MD	Public	1300
UT Health San Antonio Long School of Medicine	San Antonio, TX	MD	Public	900

**Fig. 1:**
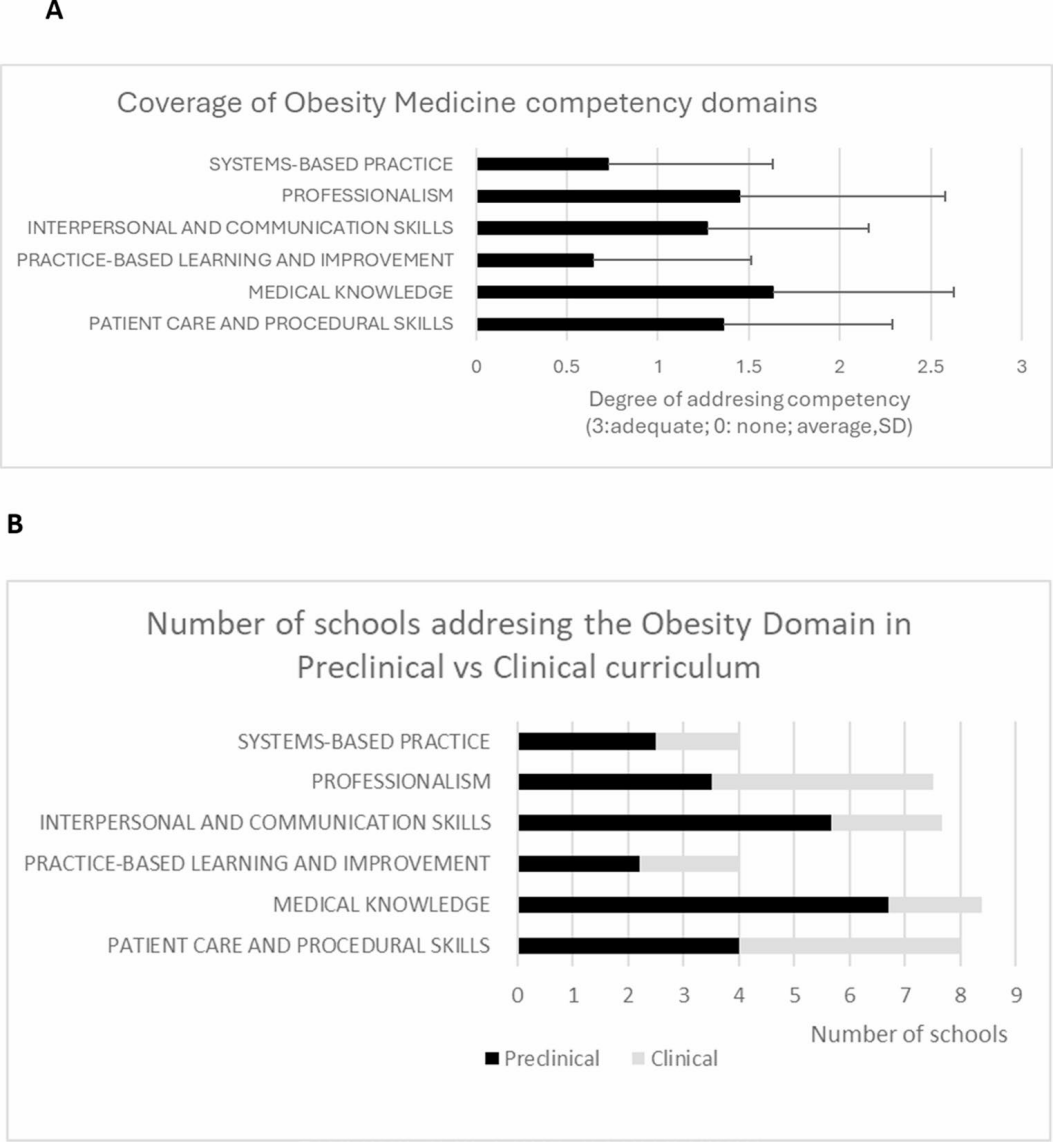
Addressing obesity medicine competencies in the curriculum. Figure **A** presents the average extent to which each obesity medicine domain is addressed in the curriculum (scored from 3 = adequately addressed to 0 = not addressed). Figure **B** presents the number of schools addressing each obesity medicine domain within the preclinical and clinical curriculum

Obesity medicine curriculum was addressed in the medical school curriculum at variate degrees (Supplemental material). The least-addressed domains were Practice-Based Learning and Improvement and Systems-Based Practice (Fig. 1A). Although the domains and competencies are covered in both the preclinical and clinical curriculum, Medical Knowledge and Interpersonal and Communication Skills are predominantly addressed in the preclinical curriculum, while Patient Care and Procedural Skills and Professionalism are taught about equally in both preclinical and clinical years (Fig. 1B). This shows a reasonable split around curriculum phases.

**Fig. 2:**
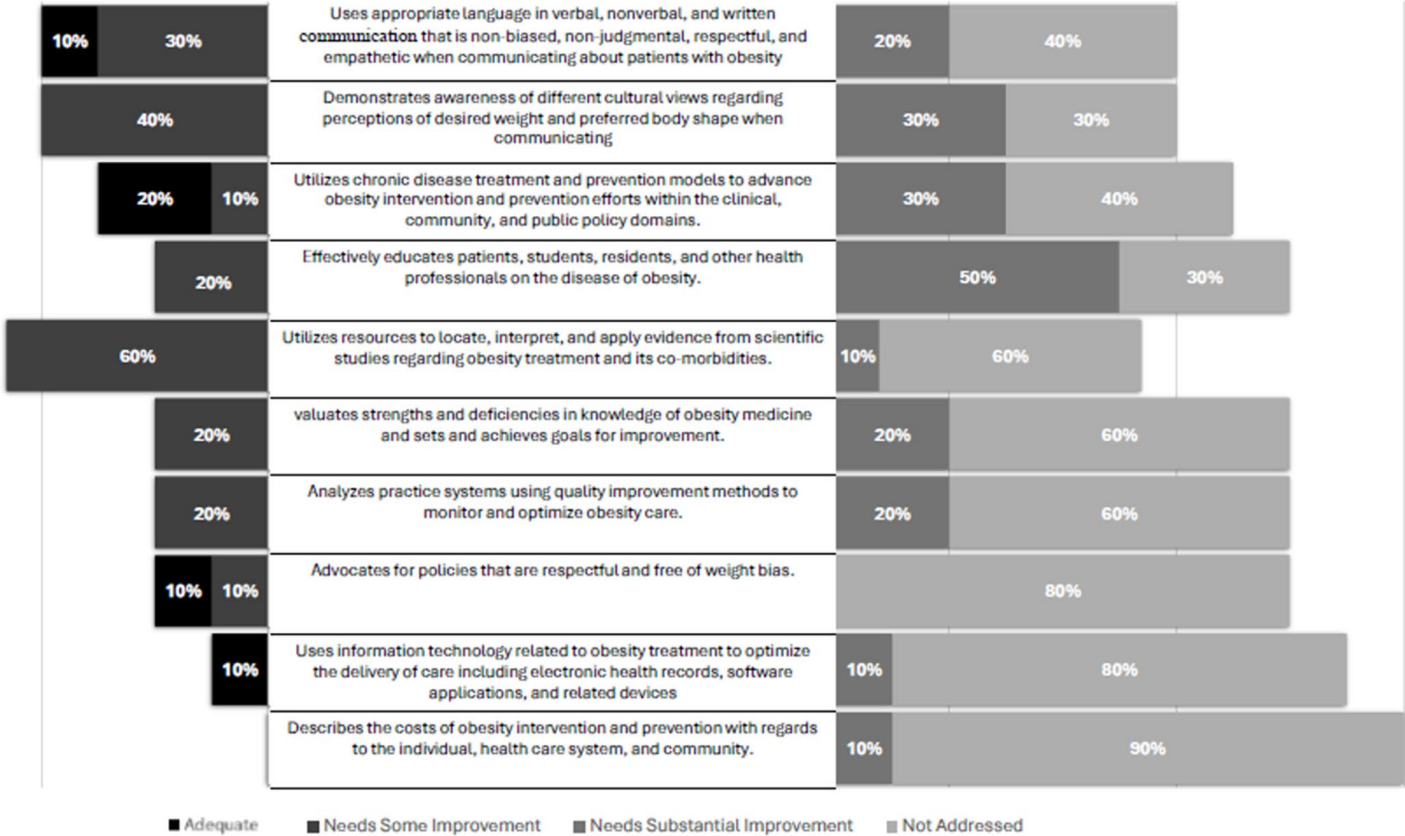
Gaps in obesity medicine curriculum by competency

Among the 32 competencies reported as either not addressed or requiring substantial improvement by at least one school, 10 were identified by 70% to 100% of schools (Fig. 2). These 10 least-addressed competencies represent the most critical targets for national-level interventions to strengthen obesity medicine education. Although many of these competencies fall within the Practice-Based Learning and Improvement and Systems-Based Practice domains, areas traditionally emphasized in graduate medical education [7], there remain important opportunities to introduce these concepts earlier in undergraduate medical education. Integrating topics such as population health, interprofessional collaboration, and experiential learning through student-run free clinics [8–11] can help bridge this gap and better prepare students for comprehensive, competency-based obesity care.

60% of the schools reported that the competencies related to “eliciting a comprehensive obesity-focused medical history” and “performing and documenting a comprehensive physical examination for the assessment of obesity” were either not addressed or required substantial improvement. Educational resources to support these competencies already exist within both preclinical and clinical curricula [12]. Strengthening these areas can be achieved by intentionally integrating obesity-focused education into clinical clerkships and longitudinal learning experiences, while providing preceptors with structured guidance and evidence-based resources to reinforce competency development in real-world settings.

50% of the schools reported that the competency “using appropriate language in verbal, nonverbal, and written communication that is nonbiased, nonjudgmental, respectful, and empathetic when communicating with patients with obesity” required substantial improvement. Several instructional strategies have been shown to effectively reduce weight stigma, including online interactive modules, independent learning activities, video vignettes, and standardized patient encounters [13–16]. Integrating these evidence-based approaches into medical curricula may help enhance communication skills and promote more compassionate, patient-centered obesity care.

**Fig. 3:**
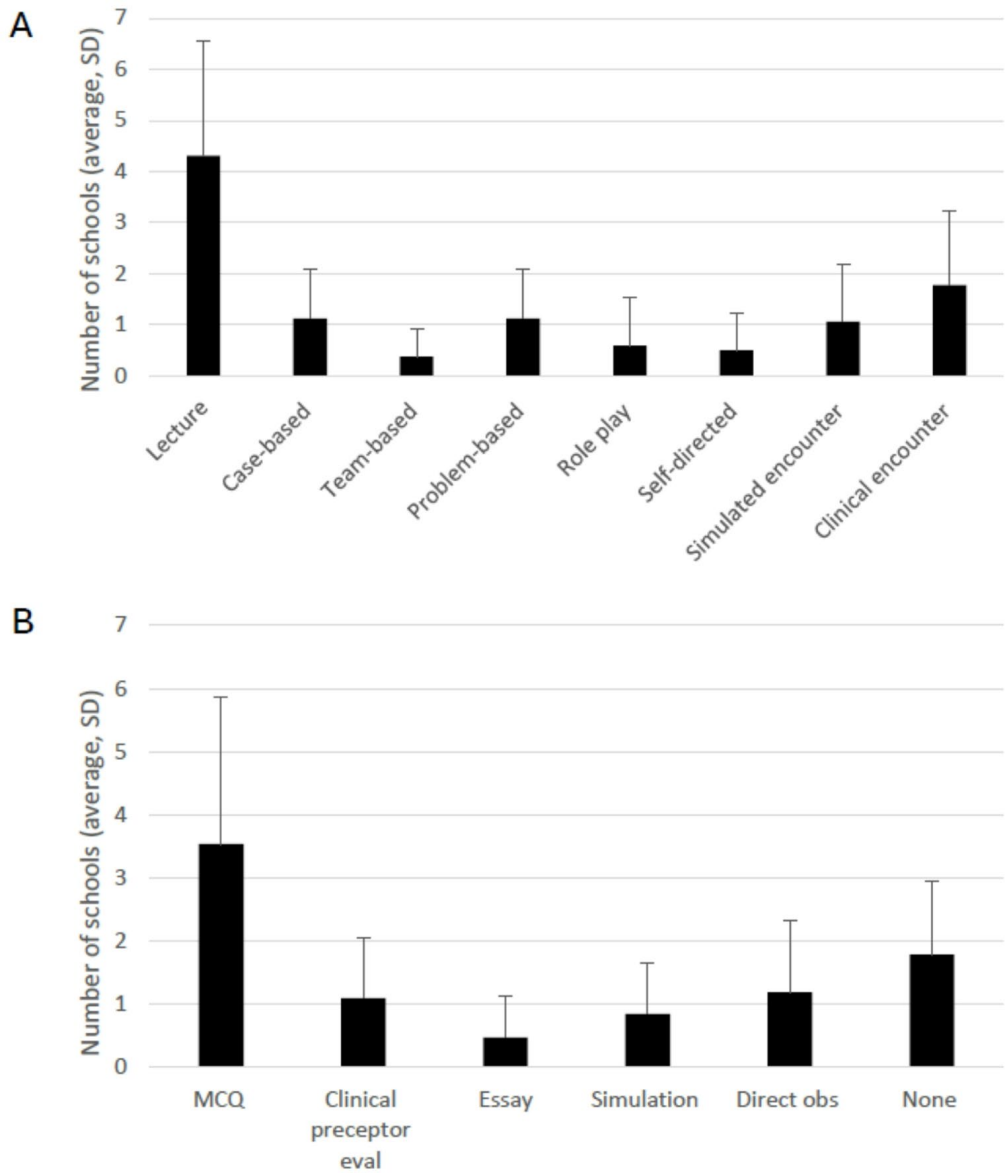
Teaching and assessment methodology for the obesity medicine curriculum

Most schools reported using lectures and clinical encounters as the primary formats for teaching obesity medicine content (Fig. 3A), while multiple-choice questions were the most commonly used assessment method (Fig. 3B). On average, two schools per competency reported not assessing the material they taught. This highlights a critical national gap in the evaluation of obesity medicine education. Addressing this deficiency will require the incorporation of more robust, evidence-based assessment methods, such as validated observed structured clinical encounters [17]to better evaluate learners’ applied knowledge, communication skills, and clinical competence in obesity care.

This needs analysis is limited by the inclusion of only ten medical schools; however, we intentionally prioritized diversity in our selection process to enhance both representativeness and relevance. This purposeful sampling ensured that the analysis reflected a wide range of institutional contexts, including variations in size, funding structure, geographic region, and curricular design. Another limitation is the reliance on self-reported data; however, this was mitigated by engaging a broad group of stakeholders within each institution’s task force, faculty, administrators, and curriculum leaders, who collaboratively completed the gap analysis survey, thereby improving accuracy and reducing individual bias. Nonetheless, the data provides baseline information to guide curriculum development aligned with the competency framework.

This national analysis reveals critical and consistent gaps in obesity medicine education across diverse medical school settings. Addressing these deficiencies is essential to equipping future physicians with the competencies needed to prevent, diagnose, and manage obesity, a leading contributor to chronic disease burden and healthcare costs. By uncovering these gaps and providing validated resources, this study provides a crucial foundation for coordinated national efforts to strengthen and standardize obesity medicine education. The results can inform a future nationwide intervention for the development of shared curricular resources, competency-based frameworks, and scalable implementation strategies that advance comprehensive, evidence-based obesity education across U.S. medical schools.

## Supplementary Information

Below is the link to the electronic supplementary material.

ESM 1 (DOCX 43.6 KB)

## Data Availability

The datasets used and/or analyzed during the current study are available from the corresponding author on reasonable request.
