# Supplementary material for: Uncovering Gaps in Obesity Medicine Competencies: Insights from Ten U.S. Medical Schools
Source: Med Sci Educ. 2026 Jan 24;36(2):539–44. doi: 10.1007/s40670-026-02646-2 (PMC13197487; doi:10.1007/s40670-026-02646-2)
Supplement: Supplementary file 1 — (DOCX 43.6 KB) [file 40670_2026_2646_MOESM1_ESM.docx]

**Supplemental Figure 1: Coverage of Obesity Medicine curriculum in US medical schools**

The figure presents number of schools reporting the 4 levels of addressing by competency. Competencies are numbered from 1-32 as below.


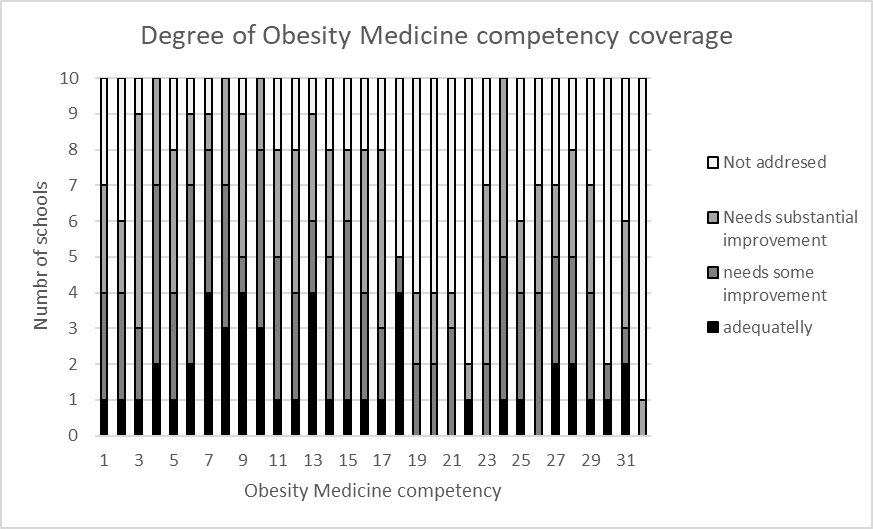


PATIENT CARE AND PROCEDURAL SKILLS

1. Elicits comprehensive obesity-focused medical history.
2. Performs and documents a comprehensive physical examination for the assessment of obesity.
3. Effectively applies clinical reasoning skills when ordering and interpreting appropriate laboratory and diagnostic tests during the evaluation of patients with obesity.
4. Utilizes evidence-based models of health behavior change to assess patients’ readiness to change in order to effectively counsel patients for weight management.
5. Engages the patients and their support systems in shared decision- making by incorporating their values and preferences in the development of a comprehensive personalized obesity management care plan.

MEDICAL KNOWLEDGE

1. Demonstrates knowledge of obesity epidemiology.
2. Demonstrates knowledge of energy homeostasis and weight regulation.
3. Demonstrates knowledge of anthropometric (body composition) measurements and clinical assessments of energy expenditure.
4. Demonstrates knowledge of the etiologies, mechanisms, and biology of obesity.
5. Demonstrates knowledge of obesity-related comorbidities and the corresponding benefits of body mass index (BMI) reduction.
6. Applies knowledge of the principles of primary, secondary, and tertiary prevention of obesity to the development of a comprehensive, personalized obesity management care plan.
7. Applies knowledge of obesity treatment guidelines to the development of a comprehensive, personalized obesity management care plan.
8. Applies knowledge of using nutrition interventions to develop a comprehensive, personalized obesity management care plan.
9. Applies knowledge of using physical activity interventions to develop a comprehensive, personalized obesity management care plan.
10. Applies knowledge of using behavioral interventions to develop a comprehensive, personalized obesity management care plan.
11. Applies knowledge of using pharmacological treatments of obesity as part of a comprehensive, personalized obesity management care plan.
12. Applies knowledge of the surgical treatments of obesity as part of a comprehensive, personalized obesity management care plan.
13. Applies knowledge of emerging treatment modalities for obesity to the development of a comprehensive, personalized obesity management care plan.

PRACTICE-BASED LEARNING AND IMPROVEMENT

1. Evaluates strengths and deficiencies in knowledge of obesity medicine, and sets and achieves goals for improvement.
2. Analyzes practice systems using quality improvement methods to monitor and optimize obesity care.
3. Utilizes resources to locate, interpret, and apply evidence from scientific studies regarding obesity treatment and its co-morbidities.
4. Uses information technology related to obesity treatment to optimize delivery of care including electronic health records, software applications, and related devices (i.e., accelerometers, resting metabolic rate and body composition analysis technology).
5. Effectively educates patients, students, residents, and other health professionals on the disease of obesity.

INTERPERSONAL AND COMMUNICATION SKILLS

1. Uses appropriate language in verbal, nonverbal, and written communication that is non-biased, non-judgmental, respectful, and empathetic when communicating with patients with obesity.
2. Uses appropriate language in verbal, nonverbal, and written communication that is non-biased, non-judgmental, respectful, and empathetic when communicating about patients with obesity with colleagues within one’s profession and other members of the healthcare team.
3. Demonstrates awareness of different cultural views regarding perceptions of desired weight and preferred body shape when communicating with the patient, family, and other members of the healthcare team.

PROFESSIONALISM

1. Demonstrates ethical behavior and integrity when counseling patients and their families who are living with overweight or obesity.
2. Displays compassion and respect toward all patients and families who are living with overweight or obesity.

SYSTEMS-BASED PRACTICE

1. Works collaboratively within an interdisciplinary team dedicated to obesity prevention and treatment strategies.
2. Advocates for policies that are respectful and free of weight bias.
3. Utilizes chronic disease treatment and prevention models to advance obesity intervention and prevention efforts within the clinical, community, and public policy domains.
4. Describes the costs of obesity intervention and prevention with regards to the individual, health care system, and community.

**Supplemental Table 1. Curriculum coverage by competency from most covered to least covered.**

| **Domain** | **Competency** | **Adequate (3)** | **Needs some improvement (2)** | **Needs substantial improvement (1)** | **Not addressed (0)** | **M** | **SD** |
| --- | --- | --- | --- | --- | --- | --- | --- |
|  |  | **No. (%)** | **No. (%)** | **No. (%)** | **No. (%)** |  |  |
| **Medical Knowledge** | Demonstrates knowledge of energy homeostasis and weight regulation | 4 (40) | 4 (40) | 1 (10) | 1 (10) | 2.1 | 0.994 |
| **Medical Knowledge** | Demonstrates knowledge of obesity-related comorbidities and the corresponding benefits of body mass index reduction | 3 (30) | 5 (50) | 2 (20) | 0 (0) | 2.1 | 0.738 |
| **Medical Knowledge** | Demonstrates knowledge of anthropometric (body composition) measurements and clinical assessments of energy expenditure | 3 (30) | 4 (40) | 3 (30) | 0 (0) | 2 | 0.816 |
| **Patient Care and Procedural Skills** | Uses evidence-based models of health behavior change to assess patients’ readiness to change to counsel patients effectively for weight management | 2 (20) | 5 (50) | 3 (30) | 0 (0) | 1.9 | 0.738 |
| **Medical Knowledge** | Applies knowledge of using nutrition interventions to develop a comprehensive, personalized obesity management care plan | 4 (40) | 2 (20) | 3 (30) | 1 (10) | 1.9 | 1.101 |
| **Medical Knowledge** | Demonstrates knowledge of obesity epidemiology | 2 (20) | 5 (50) | 2 (20) | 1 (10) | 1.8 | 0.919 |
| **Medical Knowledge** | Demonstrates knowledge of the etiologies, mechanisms, and biology of obesity | 4 (40) | 1 (10) | 4 (40) | 1 (10) | 1.8 | 1.135 |
| **Interpersonal and Communication Skills** | Uses appropriate language in verbal, nonverbal, and written communication that is nonbiased, nonjudgmental, respectful, and empathetic when communicating with patients with obesity | 1 (10) | 4 (40) | 5 (50) | 0 (0) | 1.6 | 0.699 |
| **Medical Knowledge** | Applies knowledge of using behavioral interventions to develop a comprehensive, personalized obesity management care plan | 1 (10) | 5 (50) | 2 (20) | 2 (20) | 1.5 | 0.972 |
| **Professionalism** | Displays compassion and respect toward all patients and families who are living with overweight or obesity | 2 (20) | 3 (30) | 3 (30) | 2 (20) | 1.5 | 1.08 |
| **Medical Knowledge** | Applies knowledge of the principles of primary, secondary, and tertiary prevention of obesity to the development of a comprehensive, personalized obesity management care plan | 1 (10) | 4 (40) | 3 (30) | 2 (20) | 1.4 | 0.966 |
| **Medical Knowledge** | Applies knowledge of using physical activity interventions to develop a comprehensive, personalized obesity management care plan | 1 (10) | 4 (40) | 3 (30) | 2 (20) | 1.4 | 0.966 |
| **Medical Knowledge** | Applies knowledge of emerging treatment modalities for obesity to the development of a comprehensive, personalized obesity management care plan | 4 (40) | 1 (10) | 0 (0) | 5 (50) | 1.4 | 1.506 |
| **Professionalism** | Demonstrates ethical behavior and integrity when counseling patients and their families who are living with overweight or obesity | 2 (20) | 3 (30) | 2 (20) | 3 (30) | 1.4 | 1.174 |
| **Patient Care and Procedural Skills** | Effectively applies clinical reasoning skills when ordering and interpreting appropriate laboratory and diagnostic tests during the evaluation of patients with obesity | 1 (10) | 2 (20) | 6 (60) | 1 (10) | 1.3 | 0.823 |
| **Patient Care and Procedural Skills** | Engages the patients and their support systems in shared decision-making by incorporating their values and preferences in the development of a comprehensive personalized obesity management care plan | 1 (10) | 3 (30) | 4 (40) | 2 (20) | 1.3 | 0.949 |
| **Medical Knowledge** | Applies knowledge of obesity treatment guidelines to the development of a comprehensive, personalized obesity management care plan | 1 (10) | 3 (30) | 4 (40) | 2 (20) | 1.3 | 0.949 |
| **Medical Knowledge** | Applies knowledge of using pharmacological treatments of obesity as part of a comprehensive, personalized obesity management care plan | 1 (10) | 3 (30) | 4 (40) | 2 (20) | 1.3 | 0.949 |
| **Patient Care and Procedural Skills** | Elicits comprehensive obesity-focused medical history | 1 (10) | 3 (30) | 3 (30) | 3 (30) | 1.2 | 1.033 |
| **Medical Knowledge** | Applies knowledge of the surgical treatments of obesity as part of a comprehensive, personalized obesity management care plan | 1 (10) | 2 (20) | 5 (50) | 2 (20) | 1.2 | 0.919 |
| **Systems-Based Practice** | Works collaboratively within an interdisciplinary team dedicated to obesity prevention and treatment strategies | 1 (10) | 3 (30) | 3 (30) | 3 (30) | 1.2 | 1.033 |
| **Patient Care and Procedural Skills** | Performs and documents a comprehensive physical examination for the assessment of obesity | 1 (10) | 3 (30) | 2 (20) | 4 (40) | 1.1 | 1.101 |
| **Interpersonal and Communication Skills** | Uses appropriate language in verbal, nonverbal, and written communication that is nonbiased, nonjudgmental, respectful, and empathetic when communicating about patients with obesity with colleagues within one’s profession and other members of the health care team | 1 (10) | 3 (30) | 2 (20) | 4 (40) | 1.1 | 1.101 |
| **Interpersonal and Communication Skills** | Demonstrates awareness of diverse cultural views regarding perceptions of desired weight and preferred body shape when communicating with the patient, family, and other members of the health care team | 0 (0) | 4 (40) | 3 (30) | 3 (30) | 1.1 | 0.876 |
| **Systems-Based Practice** | Uses chronic disease treatment and prevention models to advance obesity intervention and prevention efforts within the clinical, community, and public policy domains | 2 (20) | 1 (10) | 3 (30) | 4 (40) | 1.1 | 1.197 |
| **Practice-Based Learning and Improvement** | Effectively educates patients, students, residents, and other health professionals about the disease of obesity | 0 (0) | 2 (20) | 5 (50) | 3 (30) | 0.9 | 0.738 |
| **Practice-Based Learning and Improvement** | Uses resources to locate, interpret, and apply evidence from scientific studies regarding obesity treatment and its comorbidities | 0 (0) | 3 (30) | 1 (10) | 6 (60) | 0.7 | 0.949 |
| **Practice-Based Learning and Improvement** | Evaluates strengths and deficiencies in knowledge of obesity medicine and sets and achieves goals for improvement | 0 (0) | 2 (20) | 2 (20) | 6 (60) | 0.6 | 0.843 |
| **Practice-Based Learning and Improvement** | Analyzes practice systems via quality improvement methods to monitor and optimize obesity care | 0 (0) | 2 (20) | 2 (20) | 6 (60) | 0.6 | 0.843 |
| **Systems-Based Practice** | Advocates for policies that are respectful and free of weight bias | 1 (10) | 1 (10) | 0 (0) | 8 (80) | 0.5 | 1.08 |
| **Practice-Based Learning and Improvement** | Uses information technology related to obesity treatment to optimize delivery of care, including electronic health records, software applications, and related devices (ie, accelerometers, resting metabolic rate, body composition analysis technology) | 1 (10) | 0 (0) | 1 (10) | 8 (80) | 0.4 | 0.996 |
| **Systems-Based Practice** | Describes the costs of obesity intervention and prevention with regards to the individual, healthcare system, and community | 0 (0) | 0 (0) | 1 (10) | 9 (90) | 0.1 | 0.316 |
